# Subjective versus objective dental side effects from oral sleep apnea appliances

**DOI:** 10.1007/s11325-019-01852-0

**Published:** 2019-05-01

**Authors:** Marie Marklund

**Affiliations:** Department of Odontology, Medical Faculty, Umeå University, SE-906 87 Umeå, Sweden

**Keywords:** Oral appliance, Mandibular advancement device, Mandibular repositioning appliance, Mandibular advancement splint, Side-effects

## Abstract

**Purpose:**

Occlusal changes are common during long-term treatment with oral appliances (OAs) for sleep apnea. The aim of the present study was to compare subjectively reported bite changes with objective findings.

**Methods:**

Consecutive adherent treated patients were asked to participate in this study. The patients responded to two questionnaires using numeric visual analogue scales (VAS), ranging from 0 (not at all) to 10 (very much). The first questionnaire included open questions and the second questionnaire comprised specific questions about side effects. Measurements of overjet, overbite, and space for the teeth were made on plaster casts taken before treatment start and at follow-up.

**Results:**

Thirty-eight (12 women) patients with a median age of 64 years (interquartile range (IQR) 57 to 69 years) and a median treatment time of 9.5 years (IQR 5.8 to 14.3 years) were included. Overjet, overbite, the molar relationship, and the irregularity of the lower front teeth had changed significantly during treatment. There were no associations between any of the patients’ responses and the objectively measured bite changes. Younger patients, those with a small baseline overjet or overbite and those who developed an anterior crossbite were more likely to report bite changes.

**Conclusions:**

Patients who choose to continue long-term treatment with oral appliances for sleep apnea are unaware of various types of bite changes. Such changes will, however, progressively increase in magnitude and be more difficult to take care of, if needed. It is therefore important continuously to follow up patients in regard to bite changes.

## Introduction

Occlusal changes are common during long-term treatment with oral appliances (OAs) for sleep apnea [1, 2], but patients more seldom complain of any dental side effects [3–12].

**Fig. 1:**
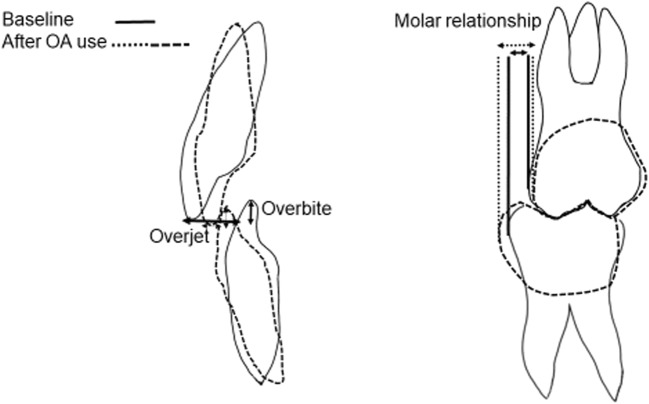
Illustration of overjet, overbite, and molar relationship measurements and expected bite change

The nightly anterior repositioning of the lower jaw with an oral appliance will produce distally directed forces on the upper teeth and anteriorly directed ones on the lower teeth [13]. The molars in the posterior parts of the dentition will reposition into a more class III relationship, and the changed inclinations of the front teeth will decrease the overjet and the overbite (Fig. 1) [1, 2]. The teeth may have more space [14–17] or become more crowded [18].

Patients less frequently report occlusal changes than objective findings reveal according to studies that have included both objective and subjective assessments [3–12]. Between 2% and 45% of patients report occlusal changes after 1 to 6 years, despite the fact that all the studies have found significant reductions in overjet and overbite [3–12]. Up to 86% of the patients have been seen to develop objectively measured occlusal changes after 5 years’ treatment, according to another study [14]. It is, however, unknown whether patients’ reports of bite changes are the same as the objectively measured findings.

The aim of the present study was to compare subjectively reported bite changes with objective findings in patients who choose to continue long-term oral appliance therapy.

## Methods

### Study design

Consecutively followed up patients from a retrospective sample answered questionnaires in order to evaluate whether patients were able to detect bite changes of various types. Patients’ reports of bite changes were compared with objective measurements on study casts. The study protocol was approved by the ethics review board at Umea University, EPN-2015/291-31, and all the patients gave their written informed consent. The sample size was estimated at least 14 patients in each group of patients who had experienced subjective bite changes and those who had not, in order to detect a difference in overjet reduction of ≥ 1 mm with a power of 0.8 and a *p* value of less than 0.05.

### Study sample

Consecutive patients who came for a follow-up and appliance replacement after at least 3 years’ treatment were asked to participate in this study. Exclusion criteria: 50% of the nights or less use of the appliance; dementia or physical illness that prevented participation in the study; insufficient quality of initial study casts; unwillingness to participate. The patients were assessed for eligibility between September and December 2015 and between March and May 2017. The patients had to be included during two time spans of consecutively followed up patients because of time constraints at the clinic.

Of 58 patients who were followed up, 20 patients were excluded. Among the excluded patients, ten reported insufficient adherence to the appliance, two patients were unable to take part because of lack of time, and one patient had no baseline plaster casts. Another seven patients were excluded, because they wanted to discontinue OA therapy and be referred for CPAP therapy; six patients because of insufficient treatment effect from OA and one patient because of increased oral mucosal tenderness when wearing the oral appliance. Thirty-eight patients were included in the study (Table 1). Among the included patients, one had received frontal restorations during the study period and another patient had molar extractions. In these two patients, it was therefore impossible to measure overjet and overbite changes or alterations in molar relationships.

**Table 1:** Baseline characteristics of the study sample (*n* = 38)

	Median (IQR)
Age (years)	64.0 (56.7–68.8)
Women (*n* (%))	12 (32)
Apnea-hypopnea index at start (*n* = 36)	10.0 (4.5–22.8)
Treatment time (years)	9.5 (5.8–14.3)
Estimated use of appliance (% of nights)	90 (81 to 92)

### Questionnaires

The patients responded to two questionnaires about adherence and side effects. The first questionnaire included open questions about side effects in order for the patients spontaneously to describe their experiences of the treatment. This questionnaire also included questions about adherence. The second specific questionnaire was distributed after the first questionnaire had been answered and given back to the dental personnel. This second questionnaire included specific questions. The patients were asked whether they had experienced bite changes of various types (Table 2). They were also asked about some other types of frequently reported side effects (Table 2).

**Table 2:** Specific questionnaire

Specific questions about bite changes, where the patients (*n* = 38) reported whether they had experienced:	*N* (%)
1. Changed bite in the morning that disappears during the day	14 (37)
2. Changed bite during the whole day	7 (18)
Bite change (1 and 2)	17 (45)
3. More irregular front teeth	6 (16)
4. More spaces between front teeth	5 (13)
5. Difficulty biting off	10 (26)
6. Difficulty chewing chewy or hard food	4 (11)
7. Used methods to minimize dental side-effects (Jig)	1 (0)
8. Has your general dentist informed you about any bite changes?	6 (16)
Questions about problems with side-effects of other types	
1. Difficulty with nose-breathing with the appliance in place	8 (21)
2. Food impaction	23 (61)
3. Teeth tenderness	21 (55)
4. Jaw tenderness	14 (37)
5. Increased salivation	14 (37)
6. Increased dryness	14 (37)

Both questionnaires used numeric visual analogue scales (VAS), ranging from 0 (not at all) to 10 (very much). A score of two or more on the scale was regarded as a subjective report of the various side effects. 

### Measurements on plaster casts

**Fig. 2:**
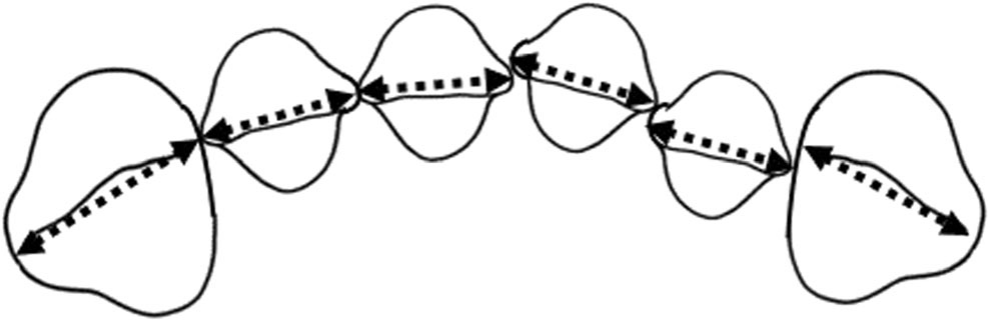
Illustration of the measurements of Little’s Irregularity Index, where the distances between the contact points (arrows) of the anterior teeth are summarized

Measurements on the plaster casts taken before treatment start and at follow-up were made regarding changes in overjet, overbite, irregular front teeth, and spacing during the treatment period. Overjet, overbite, and molar relationships were measured with a sliding caliper on the plaster casts oriented according to a wax index in central occlusion (Fig. 1). Frontal irregularity was measured with Little’s Irregularity Index [19]. This index assesses the irregularity of the front teeth. The distances between two contact points or other easily identifiable characteristics on the approximal surfaces of two adjacent front teeth are measured on all contact points between the canines. All distances are then added and described in Little’s Irregularity Index (Fig. 2). An increased value means more irregularity. Spacing between teeth was measured as the distance between two adjacent front teeth. The distances between the canines were added, separately for each jaw. The numbers of occlusal tooth contacts in the premolar-molar area on the wax indices at baseline and follow-up were registered and compared. All measurements were repeated after at least 2 weeks and blinded with respect to the results of the questionnaires. The mean value of the two measurements was used in the analysis.

### Statistical analysis

Data were described in median and interquartile ranges (IQRs). These cut-offs were used to identify patients with various bite changes and baseline characteristics. Differences in measurements before and after treatment were analyzed with Wilcoxon’s test for paired samples. The Mann-Whitney *U* test was used to compare differences between subgroups of patients. The Fisher’s exact test was used to compare patients who reported bite changes with those who did not report any changes and to identify associated characteristics. The significance level was defined as *p* < 0.05.

## Results

### Subjective reports in the questionnaires

In the first open questionnaire that was intended to show spontaneously reported side effects, 5 of the 38 patients (13%) reported occlusal changes. Other spontaneously reported problems included appliance-related problems, such as soreness or difficulty taking the device on or off in five patients (13%), dry mouth in four (11%), jaw tenderness in three (8%), periodontal problems in two (5%), or difficulty sleeping with the appliance in place in one patient (3%).

In the second specific questionnaire, 17 of the 38 patients (45%) reported occlusal changes (Table 2). These 17 patients included all five patients who had spontaneously reported occlusal changes. Only the combined  results of questions 1 and 2 were considered in the evaluations, because of the few patients who answered positively on the remaining questions.

### Objectively measured changes in dental occlusion and teeth position

Overjet and overbite decreased, the lower molars repositioned anteriorly in relation to the upper molars, and the irregularity of the lower front teeth increased in the studied sample (Table 3). The median change in overjet was − 1.6 mm (*p* < 0.001; IQR = − 2.3 mm to − 0.5 mm) and the median change in overbite was − 0.7 mm (*p* < 0.001; IQR = − 1.6 mm to − 0.1 mm) (Table 3). There was no increase in the spacing between the teeth, but the irregularity of the lower front teeth increased by 0.8 mm (*p* = 0.001; IQR = − 0.2 mm to 1.3 mm).

**Table 3:** Plaster cast measurements on occlusal changes in patients with long-term OA wear (*n* = 38)

Occlusion	Initial	At follow-up	Change	*p* value
	Median (IQR)	Median (IQR)	Median (IQR)	
Overjet (mm)^a^	3.2 (2.5 to 4.7)	2.3 (1.1 to 2.9)	− 1.6 (− 2.3 to − 0.5)	< 0.001
Overbite (mm)^a^	1.8 (1.3 to 2.6)	1.2 (0.0 to 1.9)	− 0.7 (− 1.6 to − 0.1)	< 0.001
Right molar change (mm)	1.7 (−1.7 to 2.5)	2.6 (1.3 to 3.6)	1.1 (0.6 to 2.7)	< 0.001
Left molar change (mm)^a^	2.6 (0.2 to 3.8)	3.5 1.9 to 5.4)	1.1 (0.4 to 2.1)	< 0.001
Posterior teeth in occlusal contact-right^b^	4.0 (3.0 to 4.0)	3.0 (2.8 to 4.0)	0.0 (0.0 to 0.0)	0.027
Posterior teeth in occlusal contact-left^b^	3.0 (3.0 to 4.0)	3.0 (2.0 to 4.0)	0.0 (0.0 to 0.0)	0.172
Irregularity (Little’s index)				
Upper jaw (mm)^b^	4.3 (3.3 to 6.8)	4.2 (2.6 to 6.6)	− 0.5 (− 1,0 to 0.6)	0.069
Lower jaw (mm)^a^	3.6 (2.3 to 5.3)	4.9 (2.8 to 6.3)	0.8 (− 0.2 to 1.3)	0.001
Spacing (Q 4)				
Upper jaw (mm)	0.0 (0.0 to 0.4)	0.0 (0.0 to 1.0)	0.0 (0.0 to 0.0)	0.62
Lower jaw (mm)	0.0 (0.0 to 0.0)	0.0 (0.0 to 0.0)	0.0 (0.0 to 0.0)	0.32

^a^*N* = 37

^b^*N* = 35

### Patients’ reports compared with objective findings

Five of the 17 patients who reported occlusal changes defined as 2 or more on VAS (31%) had an objectively measured overjet reduction of the median value (1.6 mm) or more, and 13 of the 21 patients who did not report any occlusal changes (62%) (*p* = 0.10) had an objective overjet reduction (Table 4). Patients’ reports of bite changes were unrelated also to changes in overbite or in molar relationships (Table 4).

**Table 4:** Reported bite changes in relation to objective findings

Cut-off at the median value	Spontaneous reports			Specific questions (Q 1 and 2)		
	Yes (*n* = 5)	No (*n* = 33)	*p* value	Yes (*n* = 17)	No (*n* = 21)	*p* value
Occlusal changes						
Large decrease in overjet (≥ 1.6 mm)	2/4	16 (49%)	1.00	5 (31%)^a^	13 (62%)	0.099
Large decrease in overbite (≥ 0.7 mm)	1/4	18 (55%)	0.340	6 (38%)^a^	13 (62%)	0.191
Large change in right molar relationship (> 1.1 mm)	4/5	15 (46%)	0.340	8 (47%)	11 (52%)	1.00
Large change in left molar relationship (> 1.1 mm)	3/5	15^b^ (46%)	0.660	7 (44%)*	11 (52%)	0.743
Initial bite characteristics						
Small initial overjet (≤ 3.2 mm)	4/4	15 (46%)	0.105	12 (75%)*	7 (33%)	0.020
Small initial overbite (≤ 1.8 mm)	3/4	15 (46%)	0.340	11 (69%)*	7 (33%)	0.049
Other background factors						
Younger (≤ 64 years)	5/5	14 (42%)	0.046	13 (77%)	6 (29%)	0.008
Females/males	1/5	11 (33%)	1.00	5 (29%)	7 (33%)	1.00
Long treatment time (> 9.5 years)	2/5	17 (52%)	1.00	8 (47%)	11 (52%)	1.00

^a^*n* = 16

^b^*n* = 32

Patients within the largest quartile of overjet reduction (≥ 2.3 mm) reported a median of 0 (IQR = 0 to 7) on VAS, while patients in the smallest quartile of overjet reduction (< 0.5 mm) reported a median of 2 on VAS (IQR = 0 to 6) (*p* = 0.78). Patients within the largest quartile of overjet reduction had a larger initial overjet of a median of 5.4 mm (IQR = 3.5 to 7.8 mm) at treatment start than the patients within the smallest quartile of overjet reduction with a median of 2.6 mm (IQR = 2.0 to 2.8 mm) (*p* = 0.002).

Four of the 38 patients had developed a negative overjet at follow-up. Two of these four patients spontaneously reported an occlusal change and three of them reported an occlusal change in the specific questionnaire.

### Characteristics of patients reporting occlusal changes

Patients who reported occlusal changes had more frequently a smaller initial overjet or overbite and were younger than those who did not report any such changes (Table 4).

## Discussion

Significant bite changes were found in the present sample of patients who had been treated in the long term with oral appliances for sleep apnea. Overjet and overbite decreased, the molars changed their relationship, and the lower front teeth became more irregular. These bite changes were not identified by the patients’ answers in the questionnaires. Patients’ reports of bite changes are therefore uncertain in follow-ups of OA treatment.

Only five patients reported spontaneously about bite changes in the open questionnaire. More patients reported bite changes in the specific questionnaire, although only 31% of these patients had an overjet reduction of 1.6 mm (median value) or more (Table 4). In the remaining group of patients who did not report any bite changes, as many as 62% also had an objectively measured overjet reduction. Consequently, the majority of the patients were unable to identify overjet reductions, despite that change in overjet is fairly easy to detect and that the patients had been informed about the possibility of bite changes developing during OA treatment. The results of the study mean that answers to both open and specific questions are uncertain when it comes to detecting objective bite changes.

Some changes in dental occlusion might be favorable [14]. Patients with a large overjet at baseline can normalize their dental occlusion during long-term treatment with oral appliances for sleep apnea. In addition, those patients with the largest initial overjet have been found to be more likely to receive a more pronounced effect of treatment than those with a smaller initial overjet [20]. This effect was also present in the present sample, since the patients with a large decrease in overjet of ≥ 2.3 mm had a larger initial overjet than those who had the smallest reductions in overjet using the device. This means that patients with the largest baseline overjet who experience the greatest benefit from an overjet reduction will also have the best chance of experiencing an effect of this kind. In summary, some patients might, in fact, be satisfied with the bite changes resulting from oral appliance therapy, since their class II malocclusion will normalize during treatment. It is therefore understandable that these patients are less likely to identify bite changes. Despite that, a large A-P bite change will reduce the advancement of the lower jaw by the appliance and there is a risk for reduced efficacy of the device, if it is left unadjusted.

Unfavorable bite changes may develop in patients who have a normal bite or class III malocclusion at baseline [14]. In these patients, long-term treatment with oral appliances may create or aggravate a malocclusion. A recent meta-analysis has concluded that the more marked the malocclusion, the more likely it is that patients will experience impaired oral health [21] and report this in a questionnaire. Three of the four patients in this study who had developed an anterior crossbite at follow-up had noticed a bite change. In addition, patients with a small baseline overjet or overbite more often reported bite changes. Consequently, the patients’ reports of bite changes are in line with dentists’ view of the severity of bite changes.

Some previous studies have reported that subjective feelings of bite changes decrease over time [6, 9], while the degree of objectively assessed bite changes increases [1–12, 16]. The patients included in the present study had used oral appliances for at least 3 years. They had chosen to continue treatment based on subjective benefits and information about effects and side effects from their dentists at earlier follow-ups, usually at intervals of 2 to 3 years. Consequently, some patients who were not included in the present study had discontinued treatment because of lack of treatment effects or dental side effects. The present sample, as well as samples in other long-term studies, might therefore primarily describe patients who are able to acclimatize easily to changes in their teeth positions or those who, in fact, benefit from the treatment. It would be of interest to study also patients who discontinue treatment. The aim of the present study was, however, to evaluate risks for undetected bite changes in patients who are positive to continue treatment.

Previous studies have used questionnaires where patients respond directly on paper [3–12] or in telephone interviews. It is not known whether the responses to the paper questionnaire were made totally beforehand, without any involvement on the part of the researchers, or were in some way influenced by the professional team. The present study primarily included a first open questionnaire in order for the patients to respond the type of side effects they had spontaneously noticed. After these responses were submitted, the patients responded to more specific questions that were thought to provide some guidance. The first questionnaire constitutes novel information on patients’ spontaneous experiences of bite changes. The very low level of positive answers in the open questionnaire compared with more frequent positive answers in the specific questionnaire in this study indicates that patients who choose to continue treatment do not seem to care significantly about bite changes.

In the question about occlusal changes, combined answers relating to permanent bite changes and temporary ones were included. In a previous study [8],only 4% of the patients had noticed permanent bite changes. This can be compared with the 41% of the patients who reported temporary bite changes, which is more in line with the 25% who had objectively measured bite changes. Fransson et al. [4] report that only 2 of 64 patients report a permanent change in occlusion, despite significant changes in overjet and overbite in the whole sample. Consequently, it might be difficult for patients to subdivide permanent and temporary bite changes and therefore both types of assessments were included.

## Conclusions

Patients who choose to continue long-term treatment with oral appliances for sleep apnea are either unaware of or do not care significantly about the various types of bite changes that may develop. These patients might therefore be at risk to suffer from undetected bite changes. It is therefore important to continuously follow up patients with respect to bite changes, since these will progressively increase in magnitude and be less easy to take care of, if needed.
